# Annotations

**Published:** 1909-11-27

**Authors:** 


### Annotations.

### "Derbyshire Neck."

Geographical nomenclature in disease is to be deprecated, for it is generally useless and often absurd to tack on to a disease the name of a place or district where it is alleged to be prevalent. "Malta fever" has lately been protested against, and now it is evident that Derbyshire should protest against the injustice that is done to that county by those who imagine that goitre is more in evidence there than anywhere else in England. Dr. Sidney Barwise, the County Medical Officer, in an interesting section of his annual report, states that the disease, so far as school children are concerned, is prevalent to the extent of about 1.5 per cent., and is to be found chiefly in the Chapel-en-le-Frith and Bakewell Unions. It has been found not only among natives, but among residents of a few years' duration. In the majority of cases—even where the diseased gland is of large size—no serious attempt has been made to treat it, and it seems to be looked upon with indifference. No case of exophthalmic goitre has been reported. The reported percentage is certainly much below the figure given in certain cantons in Switzerland or Bohemia, and is probably no larger than in Aberdeenshire or Cornwall. Statistics tell lies, but even the percentages in an annual report cannot justify the term "Derbyshire neck."

### Sleeping Sickness.

Hodges, in a recent report on sleeping sickness and the segregation camps for its treatment and observation in Uganda, comes to the conclusion that atoxyl is not so valuable a remedy as has up to the present been generally supposed. It is known, of course, that a very long time (years even) must elapse before one can say anything definitely, and as time has gone on the improvement that occurred at first in many instances has not been maintained. Very large numbers of patients have now passed through the camps in Uganda, and as they have been carefully watched and recorded they form good material for statistics. Combinations of atoxyl and mercury do not seem to have given any better results, and the outlook as regards the future treatment does not seem to be very hopeful. According to Hodges, the general type of the disease seems to have changed since the introduction of the atoxyl treatment. Paralysis, paresis, and epileptiform convulsions, which among untreated cases occurred in a small percentage, are now commoner, and may be the precursors of sudden death. Such sudden or rapid deaths, generally preceded by cerebral symptoms, also appear now to be the rule among cases that have had full doses of organic arsenic, whereas the prolonged lethargic stages which used to mark the end of untreated cases are either scarcely noticeable or absent. This is an interesting observation, as is also the case reported by Dr. C. J. Baker, of the native who, though trypanosomes were found in his blood in June 1905, was on January 1, 1909, not only alive but, as far as could be judged, in first-rate health. Apparently he had never had any treatment, and though quite well at the latter date, trypansomes were still present in his cervical glands.

### A Pamphlet on Education.

Many medical men have been circularised lately with a prospectus and leaflets directed against an alleged growth of indiscipline amongst British children of all classes. There are given the names of persons eminent in various walks of life who express approval of the objects to be furthered by a promised "Duty and Discipline" series. No doubt the writers of this are conscious of excellent intentions; and no doubt, too, there is room for improvement in the training of the young, although, as statistics bearing on crime and illiteracy show, things are steadily getting better. But we doubt whether medical practitioners have much to gain by sitting at the feet of these authors. For some time past it has appeared as if a considerable part of the adult population of Britain must be engaged in literary composition with the object of spreading various personal views; and although the printing trade must benefit, yet soon, as was wittily remarked in connection with another subject, there will not be enough disciples to go round. The sample pamphlets referred to evince no close knowledge of the subject of education, no very novel sentiments or propositions. From the literary aspect they hardly recall Rousseau's *Emile*. There is perhaps call for a "league" for promoting continence of opinion on general matters amongst, at any rate, persons frequently mentioned in the newspapers—the sole means of propaganda to be personal example.

### Tuberculosis in New South Wales.

The Sydney Government has issued a statistical analysis of the mortality from tubercular disease in the colony during the last 33 years—an analysis that differs from most medical compilations of its kind in that it has been compiled by an expert statistician. For that reason, perhaps, the figures are not so striking as they might have been, and yet they are eloquent enough if requisitioned in support of the argument that cleanliness stands pre-eminently first among our weapons against consumption. The year previous to the introduction of the Dairies Supervision Act (1886) saw the maximum tubercular mortality in New South Wales, and the passing and enforcement of the Act have unquestionably contributed to ensuring the present low mortality. Equally efficacious, it seems, was the Diseased Animals and Meat Act of 1892 in still further lowering the mortality, while the Consolidating Public Health Act of 1902 also materially added to the safeguards already possessed by the community. At present the death-rate from phthisis per 1,000 is .77 for males and .64 for females, showing a decline of 39 per cent. It is interesting to note that the percentage death-rate of peritoneal tuberculosis cases has fallen very slightly since 1886, while that of pulmonary cases which in 1886 stood at 74.7 per cent. (males) stood at 87.5 per cent. during the three years ending 1908. The compilation is a most valuable and interesting contribution to the statistics of consumption.

